# Abnormally high dislocation rate following constrained condylar knee arthroplasty for valgus knee: a case-control study

**DOI:** 10.1186/s13018-019-1325-4

**Published:** 2019-08-23

**Authors:** Feng Li, Ning Liu, Zijian Li, Kirkham B. Wood, Hua Tian

**Affiliations:** 1 Department of Orthopaedics, Peking University Third Hospital, No. 49 North Garden Road, Beijing, 100191 China; 2 Department of Orthopaedic Surgery, Stanford University Medical Center, 450 Broadway Street, Redwood City, CA 94063 USA

**Keywords:** Arthroplasty, Knee, Dislocation, Valgus, Constrained condylar knee

## Abstract

**Background:**

With the use of constrained condylar knee (CCK) prostheses, dislocation of the knee following total knee arthroplasty (TKA) with valgus deformity is rare. In our practice with such patients, however, an abnormally high dislocation rate was noted. It appeared to be associated with the extent of soft-tissue release which varied among surgeons following different sequences of release. We asked in CCK TKA with valgus deformity is releasing both the lateral collateral ligament (LCL) and popliteus tendon (PT) associated with the occurrence of dislocation.

**Methods:**

This is a case-control study of consecutive patients with valgus deformity who underwent primary CCK TKA between July 2008 and October 2015. The cases and controls were patients with and without postoperative dislocation of the knee, respectively. The extent of the release of lateral soft-tissue structures was compared between the two groups. Other patient characteristics including age, body mass index, pre- and post-operative valgus deformity, preoperative flexion-contracture, and height of the polyethylene insert were compared as well to reduce confounding.

**Results:**

Forty-three consecutive patients with a minimum 2-year follow-up were enrolled. 9.3% (4/43) of the patients had postoperative dislocation of the knee. While the dislocated patients did not significantly differ from the controls on most characteristics, they were more likely to have both the LCL and PT released together during the surgery [100% (4/4) vs. 2.6% (1/39), *P* < 0.001].

**Conclusion:**

Releasing both LCL and PT in CCK TKA with valgus deformity may increase the risk of dislocation, and need to be performed with some caution.

## Background

Achieving soft-tissue balance is a critical part of total knee arthroplasty (TKA) with valgus deformity wherein the lateral structures—the lateral collateral ligament (LCL), popliteus tendon (PT), iliotibial band (ITB), and posterolateral capsule (PLC)—are often significantly contracted [1, 2]. This situation often warrants an extensive release of these structures wherein both excessive and insufficient release can lead to postoperative instability of the knee [1–3]. Despite the development of stabilizing prosthesis such as the constrained condylar knee (CCK) [4–8], adequate lateral release remains the primary determinant of surgical outcomes [9–11].

A variety of lateral release strategies, which specify the sequence and extent of the release, have been tested in the practice of valgus TKA. However, there seems to be an open debate between two approaches regarding the following question: should LCL and/or PT be released first; or, from another perspective considering the main result of this approach, can both LCL and PT be released during the surgery? Given the primary role of LCL and PT in stabilizing the knee, most TKA surgeons would try not to [2, 4, 5, 11], yet some would [9, 12–14], especially when the lateral structures are tight in both knee flexion and extension during the surgery. The rationale of the latter is because LCL and PT stabilize the knee in both flexion and extension, releasing them first may, thus, obtain a double gain and save subsequent releases of the ITB and PLC which only function in knee extension [9].

In our practice with patients undergoing CCK TKA for valgus deformity, an abnormally high rate of dislocation of the knee was noted over the recent years. It appeared that these failed cases did not differ significantly from the other patients on most clinical parameters, with the exception that they all had both the LCL and PT released together during the surgery. To test this perceived association, the present study was performed to compare the extent of intraoperative soft-tissue release between the patients with dislocation and those without. The hypothesis was that releasing both the LCL and PT together is associated with the occurrence of dislocation following CCK TKA with valgus deformity.

## Methods

### Patient enrollment and grouping

This is a retrospective case-control study of consecutive patients in our medical record system. The eligibility criterion was patients with fixed valgus deformity of the knee who underwent primary TKA using a stemless CCK at our department. For the purpose of the homogeneity of the cases, patients receiving stemmed CCK or wedge augmentation were not included. Valgus deformity was diagnosed as one in which the center of the knee was medial to the mechanical axis of the lower extremity on the standing hip-knee-ankle radiography. The study time frame was between July 2008 and October 2015, from when our hospital first installed the present electronic medical record system to the time that allows for a minimum 3-year follow-up. Due to the rarity of bilateral CCK TKA during that period, and for the convenience of analysis, patients undergoing bilateral TKAs were excluded.

The cases and controls were defined as patients with and without postoperative dislocation of the knee, respectively. Dislocation information was obtained by reviewing the medical records including follow-up notes. For patients who were lost to follow-up, the operating surgeons interviewed them over the phone at the time of this study.

### Operative procedure and the two approaches of lateral release

The patients were operated on by two arthroplasty surgeons with similar years of experience post-training at the same department. All TKAs were performed through the regular anterior midline incision and the medial parapatellar approach. The tibial surface was resected using an extramedullary guide with the tibia in a neutral position, with a cut slope of 3°. The distal femoral surface was resected using an intramedullary guide, and the cut was set, in the coronal plane, at a valgus angle equal to the one between the anatomical and mechanical axes of the femur. Rotation of the femoral component was determined based on the epicondylar axis of the femur and Whiteside’s line. The size of the femoral component was determined using posterior referencing.

After completing the tibial and femoral osteotomies and restoring the normal alignment, the spacer block was inserted into the tibiofemoral gap to evaluate soft-tissue balance in both knee flexion and extension. The lateral supporting structures were then released accordingly using two approaches described in detail in the next paragraph. The varus-valgus instability was evaluated at 0° extension, mid-flexion (30°–40°), and 90° flexion, respectively, to determine the type of insert needed: a CCK insert was used when the knee was considered unstable—mediolateral gap asymmetry greater than 3 mm—in any of these positions even if tensioned with a spacer bar or a trial posterior stabilized component. After installing the insert, varus and valgus stresses were applied again to ascertain the stability of the knee. In this series, all patients were implanted with the Genesis II™ posterior-stabilized total knee system (Smith & Nephew, Memphis, TN). The inserts were made of standard cross-linked polyethylene (PE). All components were cemented using the gentamicin-containing PALACOS cement (Heraeus Medical Gmbh, Germany). The patella was processed by resurfacing or patellaplasty to secure a normal patellofemoral movement. Retinacular release was performed when the patella was subluxed laterally as assessed by the “no-thumb” test using trial components.

The release of lateral structures was performed dependent upon surgeon preference. It was consistently started with the ITB and PLC when the lateral structures were tight in knee extension only. However, when the lateral structures were tight in both knee flexion and extension, the surgeons’ maneuvers diverged: one still released ITB and PLC first and then, if needed, released LCL but tried to leave the PT intact; the other surgeon instead released the LCL or PT or both first (from their attachments to lateral femoral condyle), followed by ITB and PLC if needed.

### Post-operative management

Drainage and antibiotics were withdrawn within 48 h. Low molecular weight heparin and a mechanical foot-pump system were administered to prevent deep vein thrombosis (DVT). The patients were immediately instructed to begin in-bed static quadriceps and active plantar flexion/extension exercises and were advised to perform active knee extension and straight-leg raise exercises after drain removal. Patients were generally followed up at 3 months, 1 year, and then annually postoperatively.

### Outcomes and measures

The cases and controls—patients with and without dislocation—were compared on (a) general clinical characteristics including sex, age, BMI, preoperative flexion contracture, pre- and post-operative valgus, and the height of the PE insert, and (b) the extent of the lateral release. The percentage of patients who had both the LCL and PT released during surgery was compared between the two groups.

All radiological parameters were measured in degrees using the hip-knee-ankle anteroposterior (AP) and lateral views preoperatively and at final follow-ups. For patients with dislocation, the postoperative parameters were measured at the last follow-up before dislocation. The extent of the soft-tissue release was obtained from the operation notes. Information regarding the dislocations, as well as other complications in all patients, was summarized as well.

### Statistical analysis

Descriptive statistics were reported in mean with range or median with 25% and 75% quantiles (IQR). The general clinical characteristics of the patients with and without dislocation were plotted and compared visually. Fisher’s exact test was used to compare percentages. R version 3.4.3 software (R Foundation for Statistical Computing) was used for data analysis. The α value was set at 0.05.

## Results

Forty-three patients (39 women and 4 men) who underwent primary CCK TKA for a valgus knee were finally enrolled. They had a median age of 65 years (IQR 60, 71.5), a median BMI of 25.2 (IQR 24.5, 26), and an average follow-up time of 5 years (2~10 years). Their diagnoses include osteoarthritis in 33 patients, rheumatic arthritis in 7, psoriatic arthritis in 2, and traumatic arthritis in 1. Their median preoperative flexion contracture was 10^°^ (IQR 0, 20^°^). In all patients, the median valgus deformity was corrected from 14° (IQR 11^°^, 17.5^°^) preoperatively to 0^°^ (IQR 0, 3.5^°^) at the final follow-up.

**Fig. 1:**
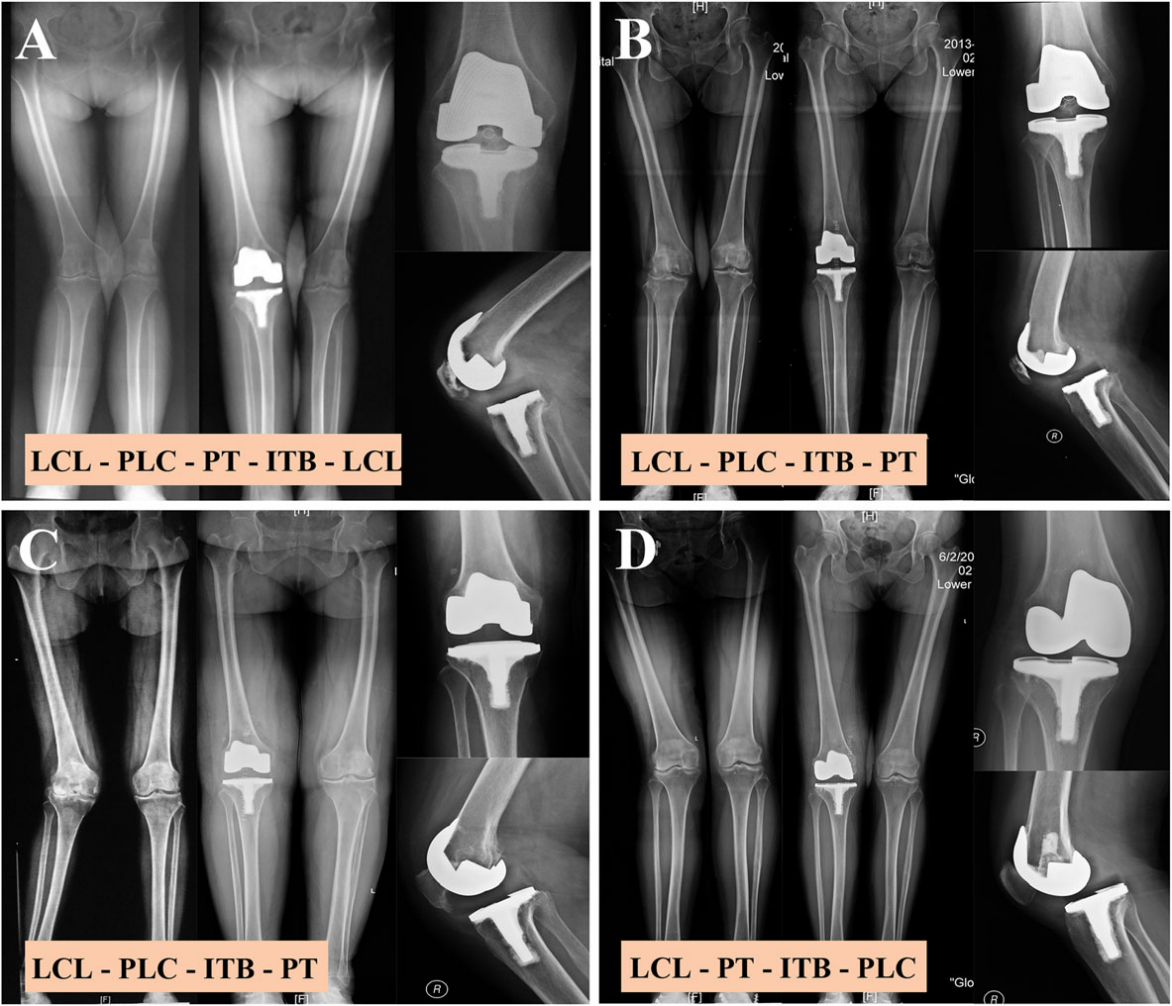
Radiographs of the dislocated patients showing the structures released and the release sequence. **a**, **b**, **c**, and **d** represent patients 1, 16, 29, and 34 in Table 1, respectively. The release sequence was shown for each patient. ITB stands for the iliotibial band; PLC, posterolateral capsule; PT, popliteal tendon; LCL, lateral collateral ligament

**Fig. 2:**
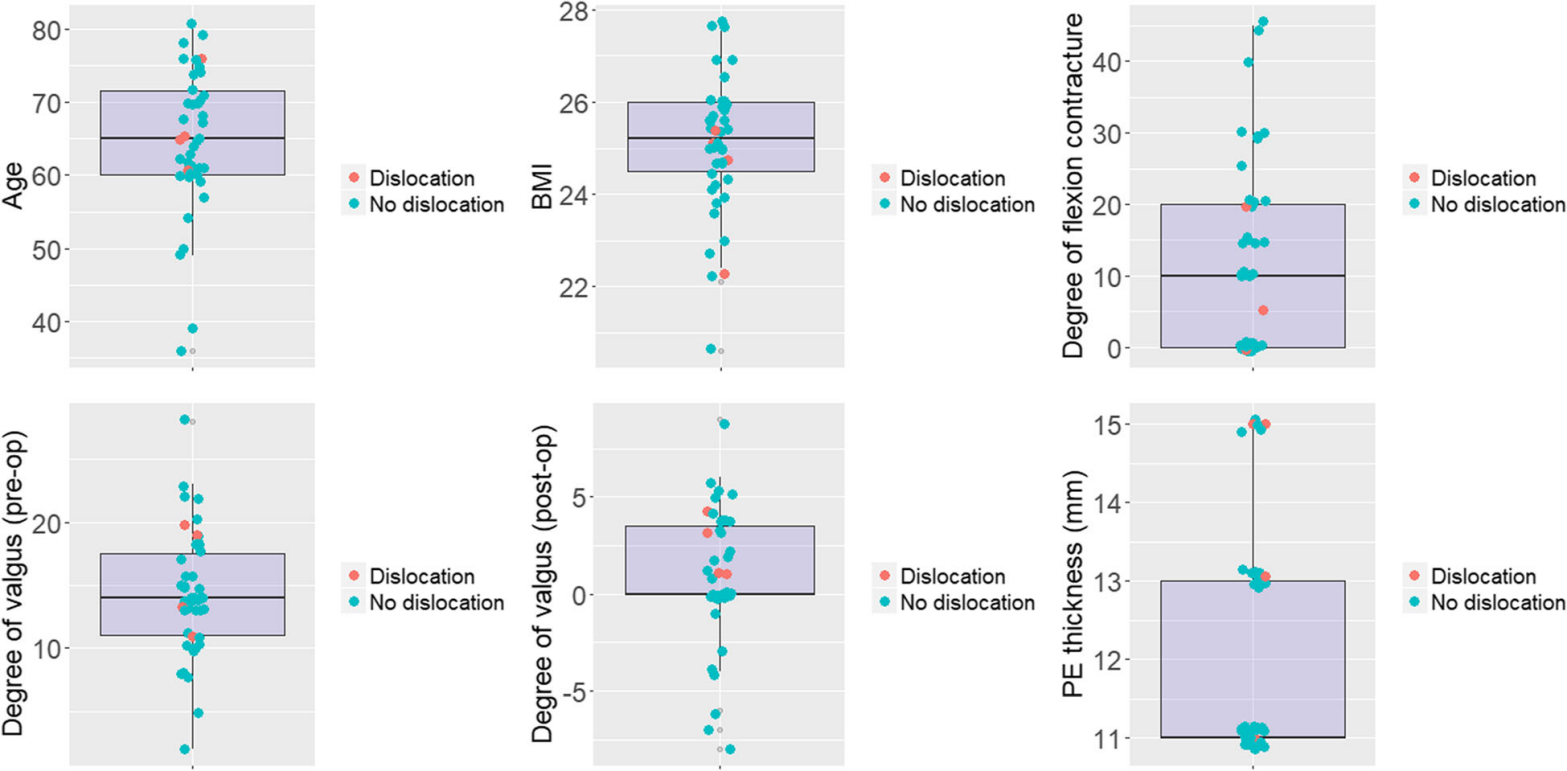
Comparing demographics and deformity parameters between patients with and without dislocation after CCK TKA. The pink points represent patients with dislocation while the blue ones represent those without, both plotted against the boxplot showing the median (the thickened horizontal line) and quartiles. CCK stands for constrained condylar knee; TKA, total knee arthroplasty

**Fig. 3:**
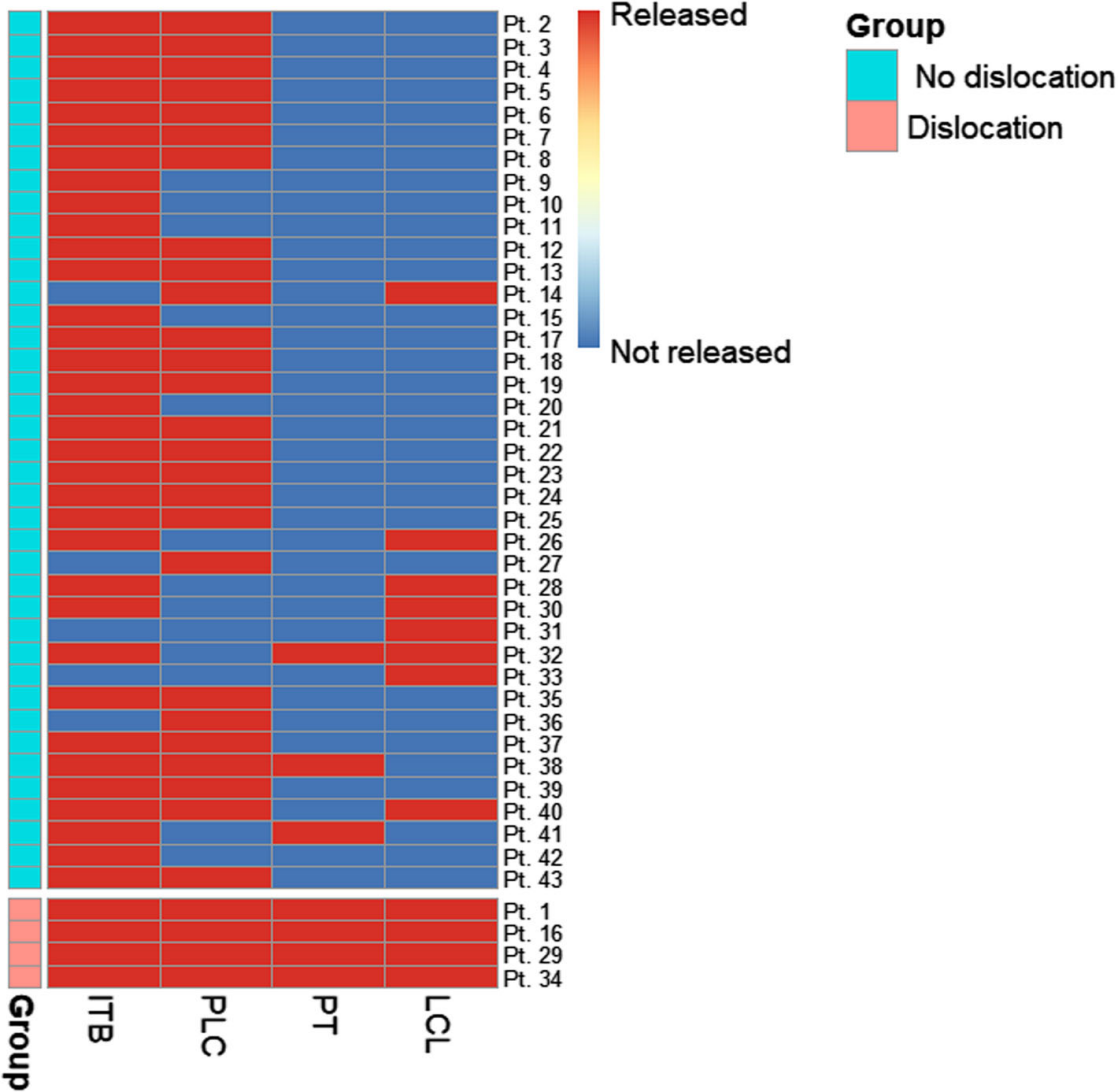
The lateral structures released in patients with and without dislocation of the knee following CCK TKA. A blood-red rectangle indicates that the structure was released and a blue one, not released. CCK stands for constrained condylar knee; TKA, total knee arthroplasty; ITB, iliotibial band; PLC, posterolateral capsule; PT, popliteal tendon; LCL, lateral collateral ligament

9.3% (4/43) of the patients had a postoperative dislocation of the knee. Their clinical and radiological information was summarized in Table 1 and Fig. 1, respectively. These dislocated patients did not differ from the controls on most clinical characteristics (Fig. 2). However, they were more likely to have both the LCL and PT released during surgery [100% (4/4) vs. 2.6% (1/39), *P* < 0.001, Fig. 3].

**Table 1:** Clinical information of the four dislocated patients

Patient ID	Age	Time of dislocation post-operatively	Trigger of dislocation	Treatment for dislocation
1	76	4 years	Trying to arise from a low chair	Revision surgery
16	65	37 days	Trying to arise from sitting cross-legged in a chair	Closed reduction
29	65	2.5 years	Trying to arise from a low chair	Firstly underwent closed reduction at home by her physician son, then had radiographs taken and underwent revision surgery.
34	61	2 years	Trying to arise from sitting cross-legged on the floor	Closed reduction

*All were female diagnosed with osteoarthritis of the knee. Radiographs of dislocations are shown in Fig. 1

The four patients with dislocation did not have other complications. In patients without dislocation, the complication rate was 12.8% (5/39), and included DVT in 2 patients, medial collateral ligament injury in 1, foot drop due to peroneal nerve injury in 1, and periprosthetic fracture in 1.

## Discussion

In this study, we reported a high dislocation rate of 9.3% in our primary CCK TKAs in patients with valgus deformity and found that the dislocation was associated with the release of both LCL and PT. This association was further corroborated by the finding that the patients with and without dislocation did not differ significantly on other clinical characteristics.

From the operation notes, the reason that both LCL and PT were released in the dislocated patients is that they (one or both) were released before ITB and PLC (Fig. 1). As LCL and PT stabilize the knee in both flexion and extension [13], this approach was used when lateral tightness of the knee was noted in both positions during surgery and aimed at balancing the knee in both positions simultaneously. One theoretical merit of this approach is that it may, thus, save subsequent releases of the ITB and PLC which only function in knee extension [9]. Also, previous researches have demonstrated that releasing LCL and/or PT first can be safe and effective. In a series of 189 valgus TKAs performed this way, satisfying deformity correction was achieved without any postoperative instability issues or revision surgeries at 6-year follow-up [9].

Two factors could help to explain the mechanism whereby releasing LCL and/or PT first, in our hand, may have led to the dislocations. Firstly, the initial releasing of LCL and/or PT has consistently led to subsequent releases of the other structures and, in one patient, a re-release of LCL (Fig. 1), all of which increased the risk of instability. This suggested that releasing LCL and/or PT first in valgus TKA is technically demanding and may have a risky learning curve. Also, with the use of CCK, an intrinsic pursuit for “perfect” soft-tissue balance may have influenced our surgical maneuver towards more liberal releasing of soft tissues.

From a biomechanical perspective, LCL and PT are two main stabilizing structures of the knee, especially in knee flexion [1]. In cadaveric TKAs, the lateral joint gap generated by releasing these two ligaments typically shows an exponential increase as compared to that generated by release of the ITB or PLC: in lateral release performed in the sequence described by Insall et al. [11], while releasing ITB produced a lateral gap increase of less than 3 mm, a subsequent release of LCL and PT further increased the gap by 3.8 times [1]. These results suggest that releasing the LCL and PT is more susceptible to over-releasing which can lead to instability problems including dislocation after TKA. From our results, such dislocations are typically incurred by certain movements of the knee, such as rising from a low chair or from sitting cross-legged (Table 1), wherein the dislocating force can be significant and greater than that applied to ascertain stability during the surgery.

CCK per se has been shown to be effective and reliable in most valgus TKA with rare instability complications. In a series of 49 primary TKA patients (55 knees) with valgus greater than 15°, stemless CCK resulted in improved function and pain relief; no instability or radiographic loosening was noted during a 2–6-year follow-up [5]. Only one patient had a chronic patellar dislocation. In a survival analysis of 184 TKAs using stemless CCK of which the majority were valgus knees, the 5-year survival rate of the prosthesis was 97.3%: the 14 revisions included implant loosening in 7 knees, infection in 2, stiffness in 4, implant fracture in 1, but did not have any dislocations [6]. Another study reported the 10-year outcomes of 54 primary TKAs (44 patients) receiving CCK of which at least 28 had valgus deformity: the 10-year prosthesis survival rate was 96%; there was only 1 posterior dislocation and 2 patients with implant loosening; no other revision surgeries were reported [4]. A study focusing on valgus TKA also reported 0 dislocation in 105 CCK TKAs in a minimum 7-year follow-up, although there were 2 cases with aseptic loosening [15]. Given the rarity of dislocation following CCK TKA in literature, the dislocations in this series were less likely to be related to the CCK prosthesis per se.

Considering the abnormally high rate of dislocation in this series, we now would try not to release both LCL and PT together in TKA with valgus knee and would release ITB and PLC first. For patients undergoing extensive lateral release and are, therefore, at risk of the instability of the knee, using a more constrained prosthesis such as the stemmed CCK is considered. Also, postoperatively, such patients are advised to avoid certain postures that may increase the likelihood of dislocating, especially sitting cross-legged on the floor, for at least 3 months and followed up by both the arthroplasty surgeon and a physical therapist associated with the arthroplasty service.

This study is limited by its retrospective design and small sample size. The treatment and prognosis of the dislocated patients were not studied in detail. As a single-institution study, the results are biased by local risk factors such as surgeon performance. However, as our institution is a tertiary teaching hospital, the finding of this study remains suggestive of the increased risk of dislocation from releasing both LCL and PT together or, at least, a learning curve for doing so.

## Conclusions

In conclusion, our results suggest that releasing both the LCL and PT together in CCK TKA with valgus deformity may increase the risk of dislocation and, therefore, may need to be performed with some caution. Future studies with a prospective design and larger sample size are needed to lend more credence to our results.

## Data Availability

Upon request, raw data can be provided.

## Abbreviations

BMI: Body mass index
CCK: Constrained condylar knee
ITB: Iliotibial band
LCL: Lateral collateral ligament
PLC: Posterolateral capsule
PT: Popliteus tendon
TKA: Total knee arthroplasty
